# Supplementary material for: Mechanisms underlying pathological cortical bursts during metabolic depletion
Source: Nat Commun. 2023 Aug 8;14:4792. doi: 10.1038/s41467-023-40437-0 (PMC10409751; doi:10.1038/s41467-023-40437-0)
Supplement: Supplementary file 1 — Supplementary Information [file 41467_2023_40437_MOESM1_ESM.pdf]

Supplementary information

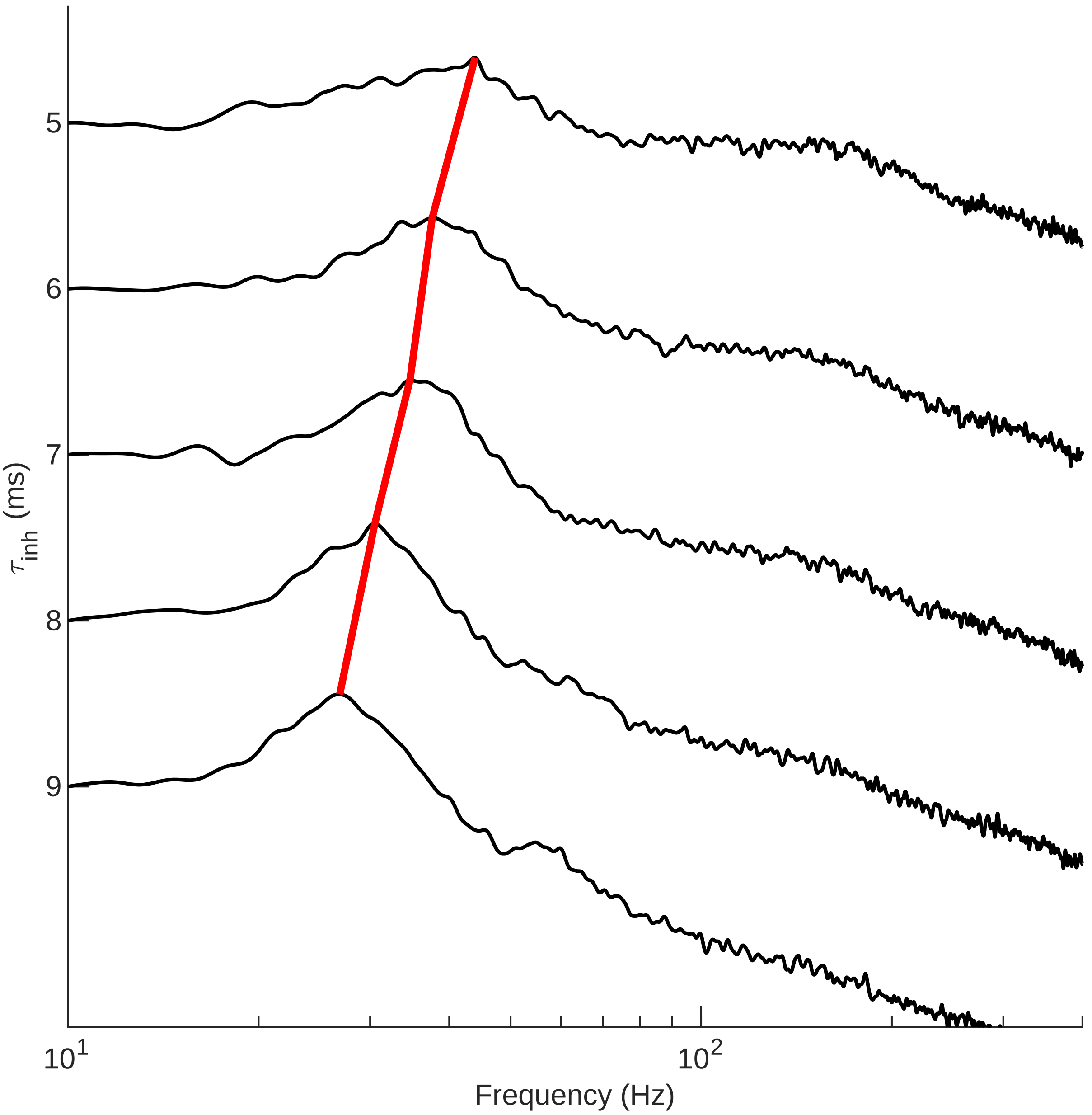

Supplementary Figure S1: Dependence of AI state network oscillatory frequency on inhibitory time constant. Curves show log(power spectrum) for a series of values of τ_{inh}. Red line joins the spectral peaks to illustrate the relationship between the peak frequency and τ_{inh}.

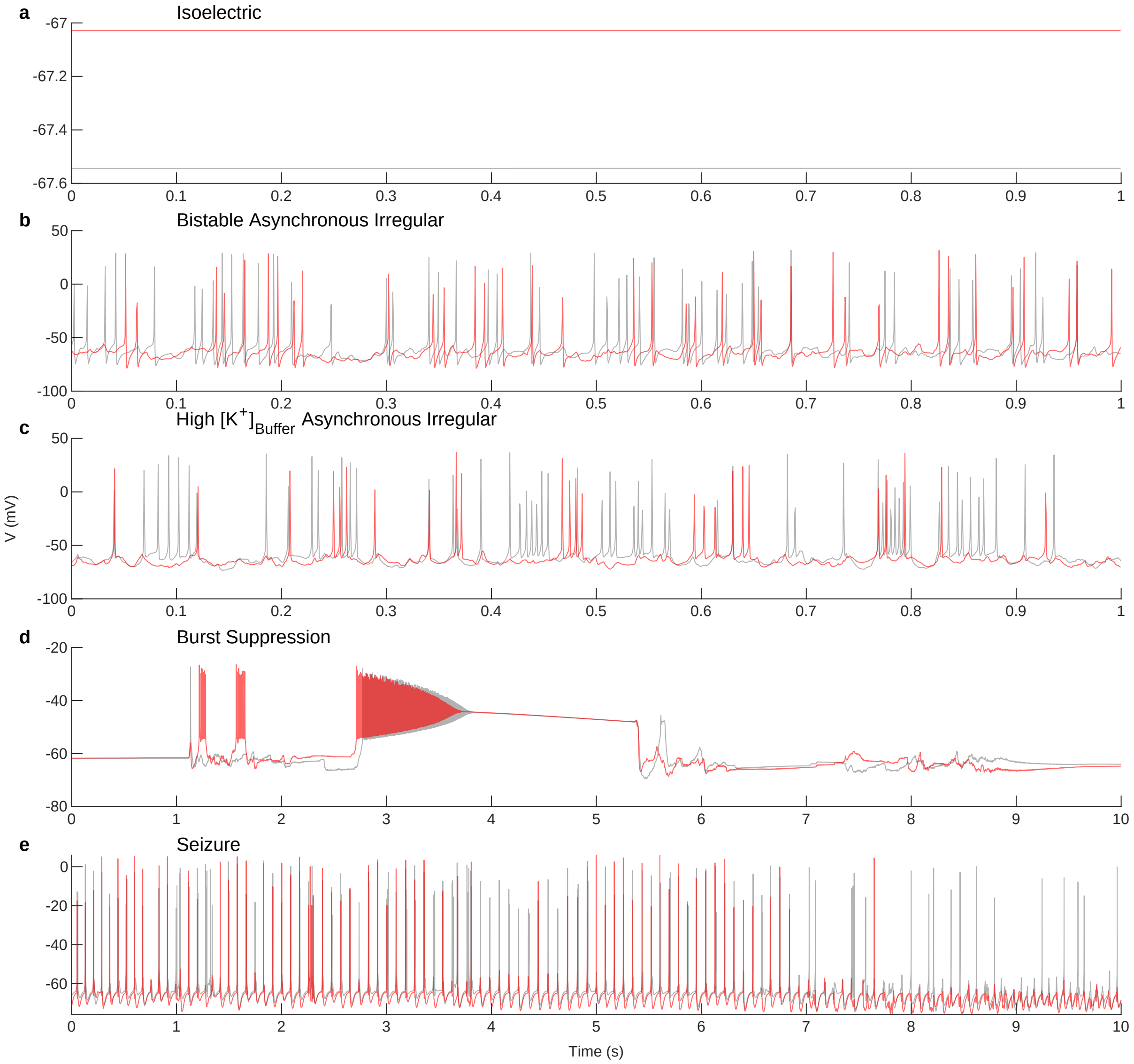

Supplementary Figure S2: Example membrane potential of a single excitatory neuron (black) and single inhibitory neuron (red) for different states. a, Isoelectric. **b**, Bistable Asynchronous Irregular. **c**, High $[K^+]_{Buffer}$ Asynchronous Irregular. **d**, Burst Suppression. **e**, Seizure, marked by synchronicity between the selected excitatory and inhibitory neurons. This panel illustrates the dynamics of two neurons during a seizure state, but it should be noted that a full seizure event involves widespread synchronicity across an ensemble of neurons, which is better captured at the network level (see Fig. 6 in the main text).

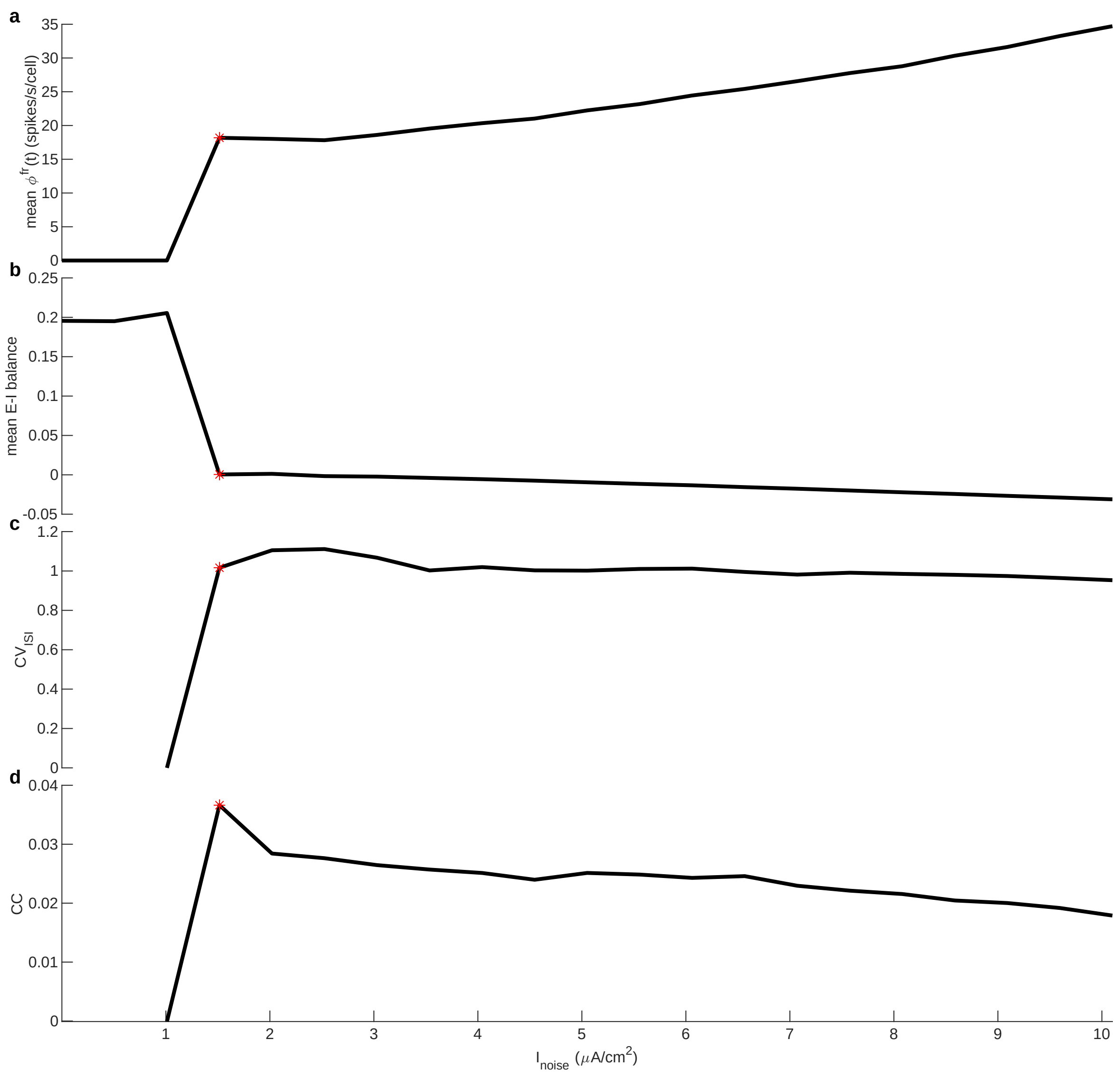

Supplementary Figure S3: Stochastic noise transitions the bistable regime from isoelectric to AI. **a**, Starting from initial conditions in the isoelectric state, a weak stochastic input current of amplitude sim 1.5 /(μA/cm^2) (red asterisk), or higher, yields spiking dynamics. **b-d**, Further analyses show that the mean E-I balance (**b**), coefficient of variation (**c**), and correlation coefficient (**d**) rapidly converge to values consistent with the noise free AI state.

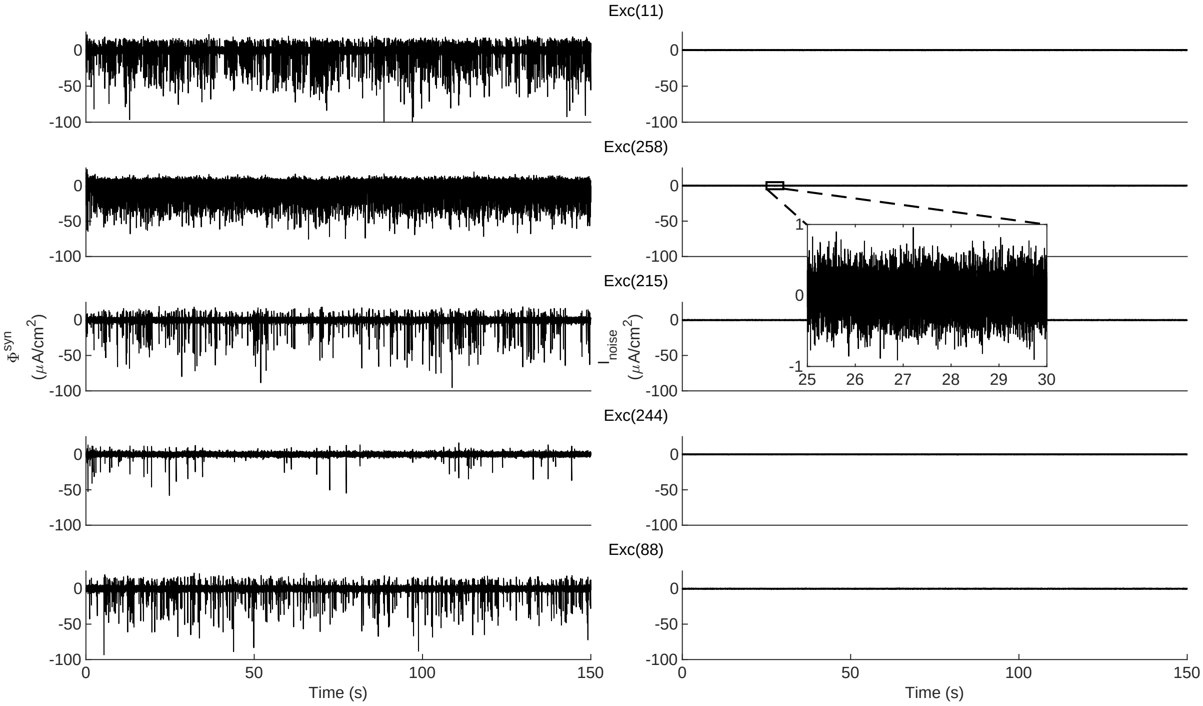

Supplementary Figure S4: Comparison of representative post-synaptic currents (Φ^{syn}, left panels) and external stochastic currents ($\mathbf{I_{noise}}$, right panels) sufficient to perturb the system away from the isolelectric state during physiological values of $[\mathbf{K}^+]_{\mathbf{Buffer}}$ and $[\mathbf{O_2}]_{\mathbf{Buffer}}$. Each row represents an excitatory neuron picked at random: Excitatory neurons numbered 11, 258, 215, 244, and 88. The inset in the right panel is the zoom showing 5 s of $\mathrm{I_{noise}}$ for excitatory neuron number 258.

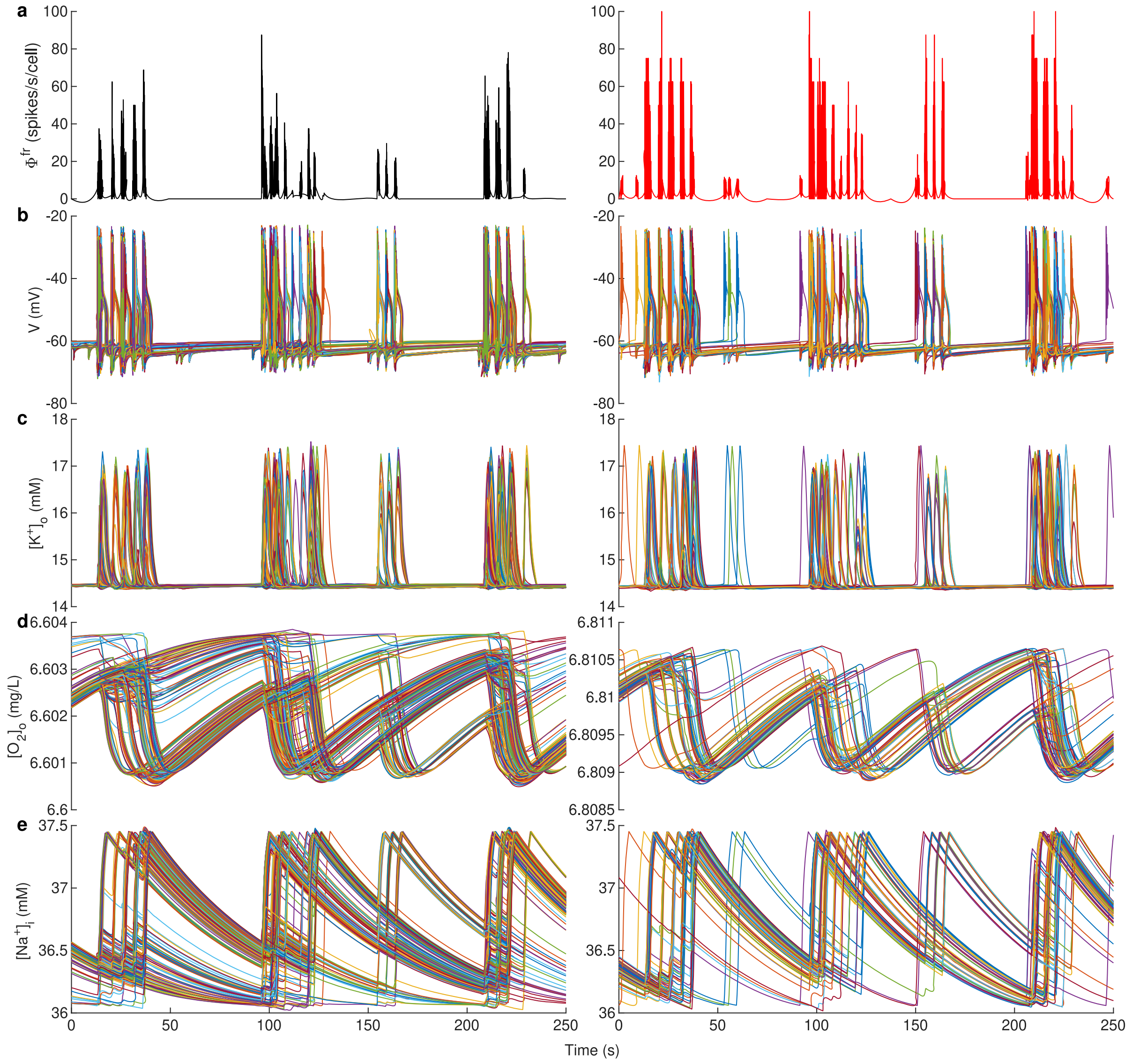

Supplementary Figure S5: Individual state variables of all neurons (left:excitatory; right:inhibitory) during the BS state. **a**, Average firing rate. **b**, Membrane voltage of individual neurons. **c**, Extracellular potassium of individual neurons. **d**, Extracellular oxygen of individual neurons. **e**, Intracellular sodium of individual neurons.

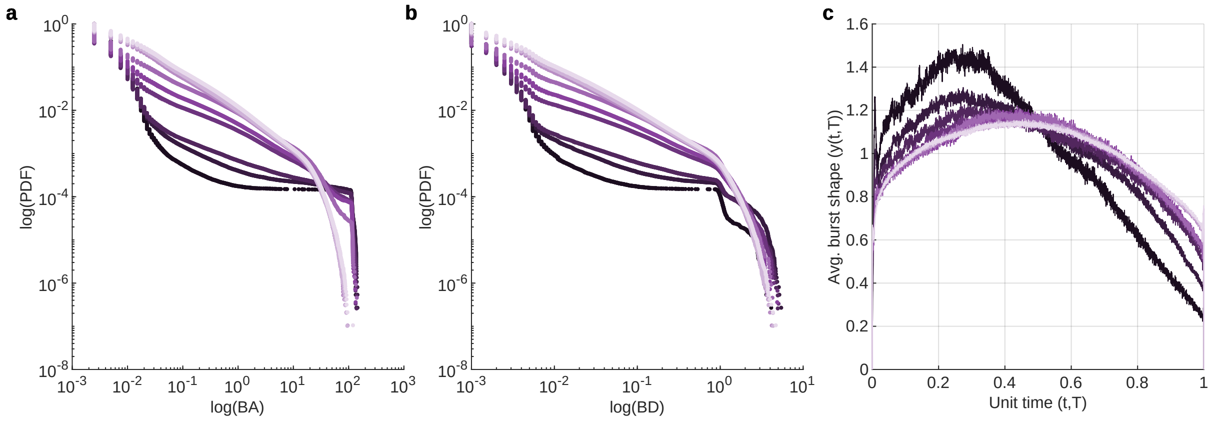

Supplementary Figure S6: **a**, Burst area upper cumulative distributions P(BA) of different examples from $[O_2]_{\mathrm{Buffer}} \in [6.5, 10]$ mg/L displaying wider range of bursts including ordered, scale-free, and disordered bursts shown as shades of purple. **b**, Corresponding burst duration upper cumulative distributions P(BD). **c**, Corresponding average burst shapes. Average is over all bursts of duration 1280 ms to 5120 ms.

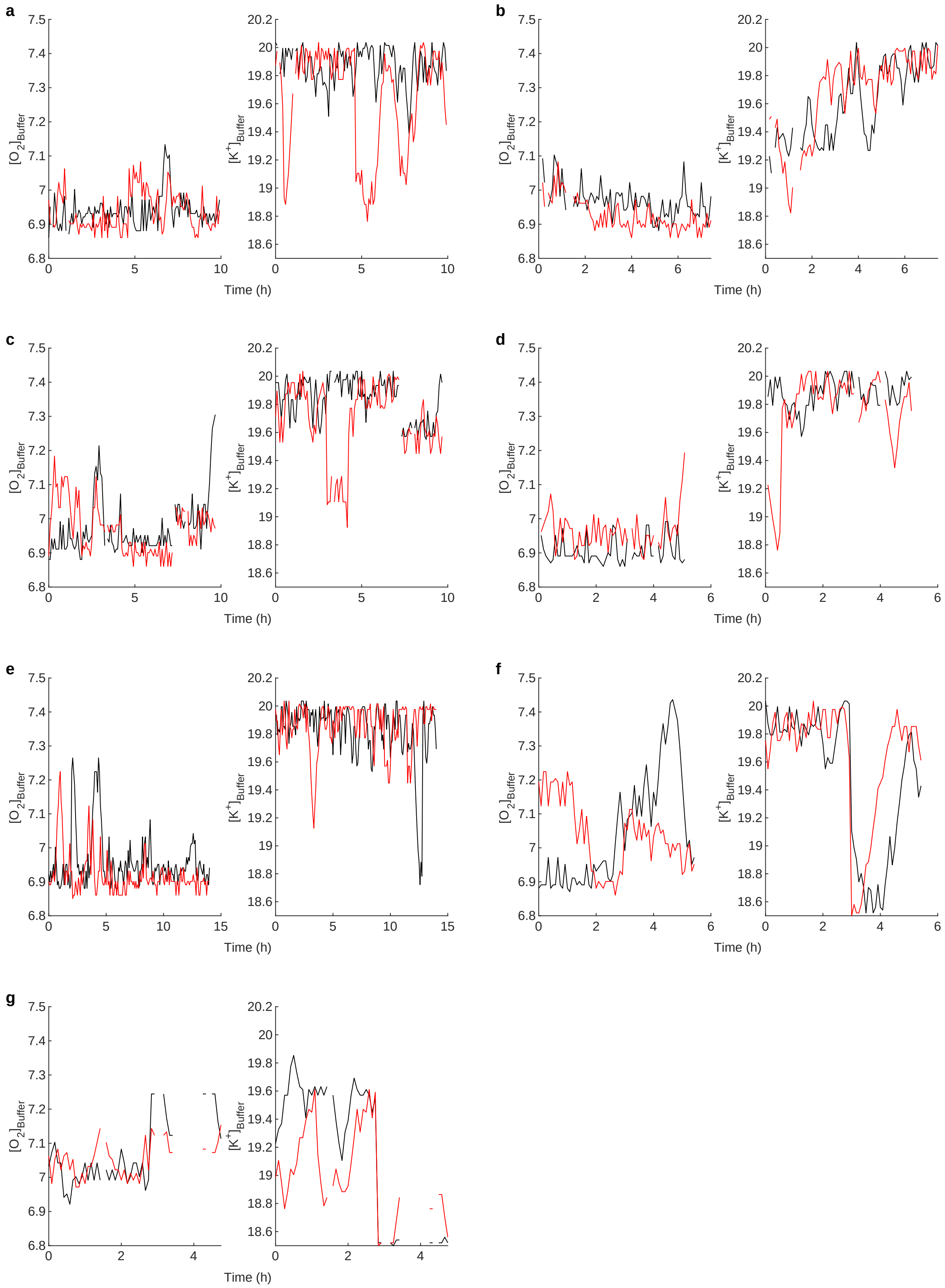

Supplementary Figure S7: Comparison between metabolic parameters inferred from the firing rate time series (original, black) and from the instantaneous power of postsynaptic current time series (red) for infants with poor recovery outcomes. **a-g**, Infants 1-7.

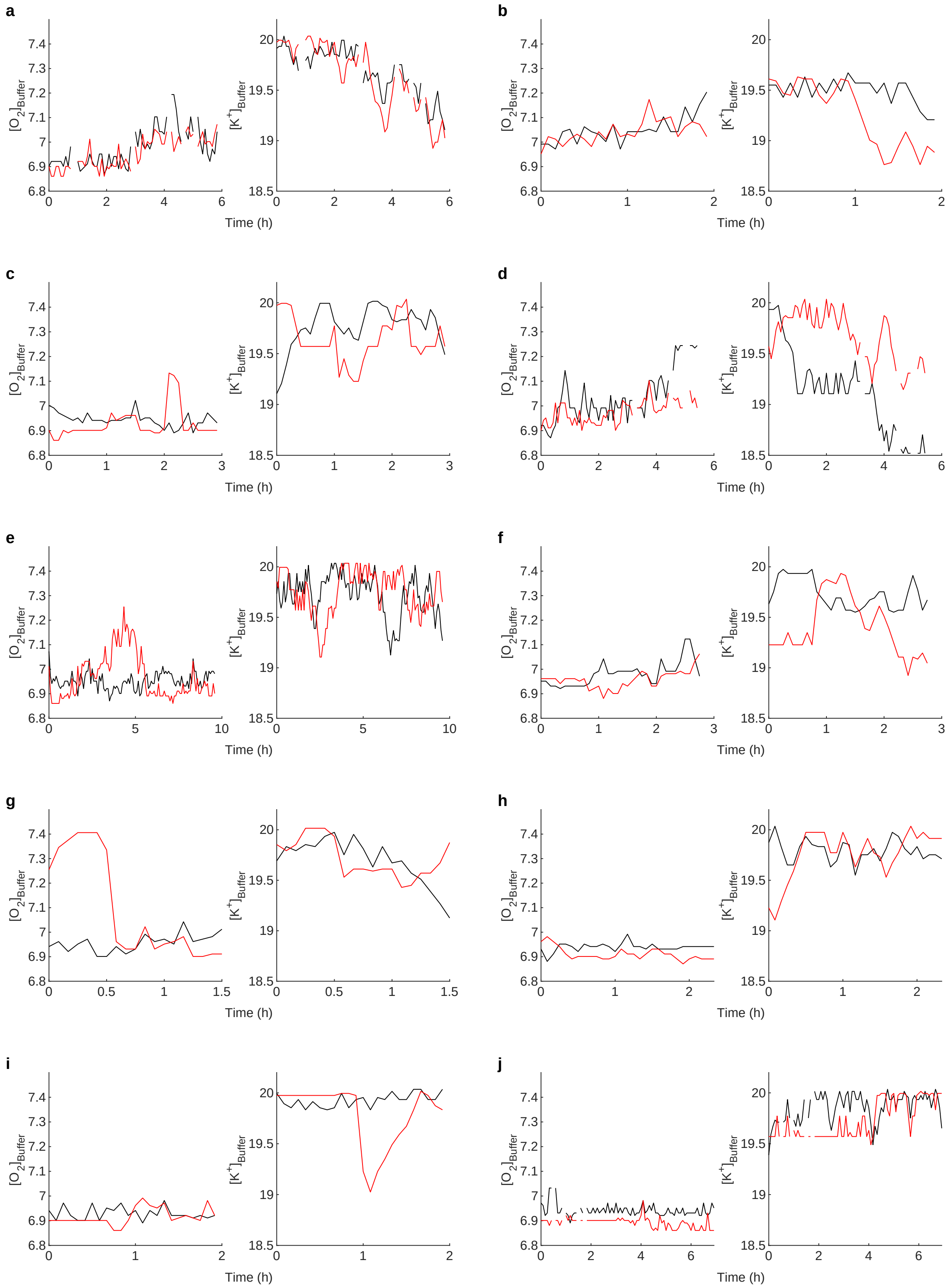

Supplementary Figure S8: Comparison between metabolic parameters inferred from the firing rate time series (original, black) and from the instantaneous power of postsynaptic current time series (red) for infants with good recovery outcomes. **a-j**, Infants 1-10.

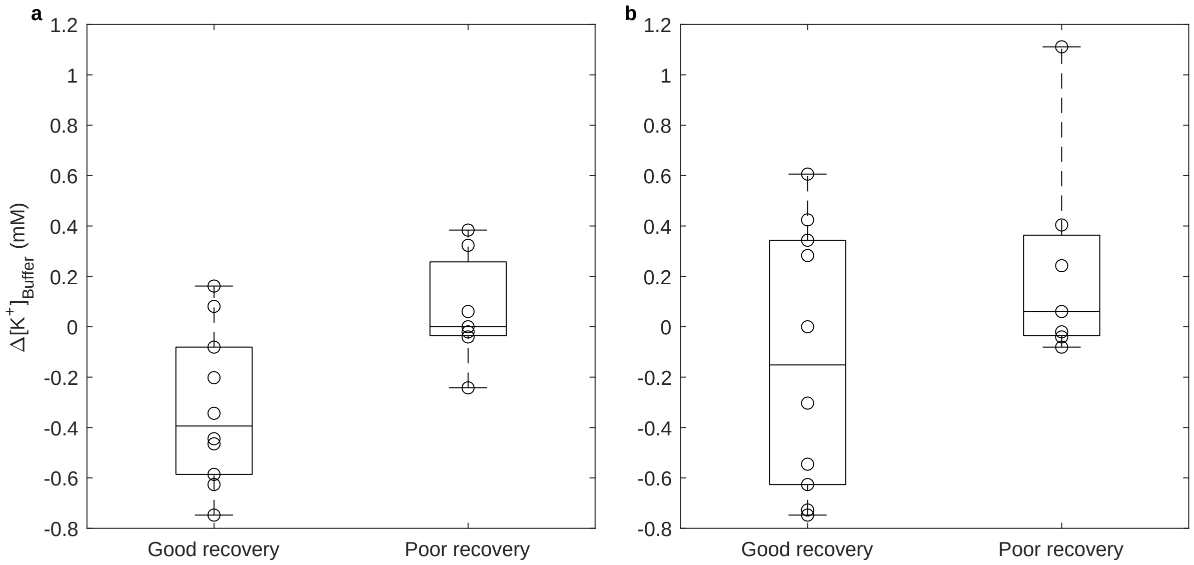

Supplementary Figure S9: Box and whisker plots for $\Delta[K^+]_{Buffer}$ from the last epoch for infants with good (n=10) recovery outcome versus poor recovery outcome (n=7). **a**, Using firing-rate time series as the LFP proxy. **b**, Using instantaneous power of the postsynaptic currents time series as the LFP proxy. Circles, all individual (per infant) $\Delta[K^+]_{Buffer}$ values; center line, median; box limits, upper and lower quartiles; whiskers, minimum and maximum.

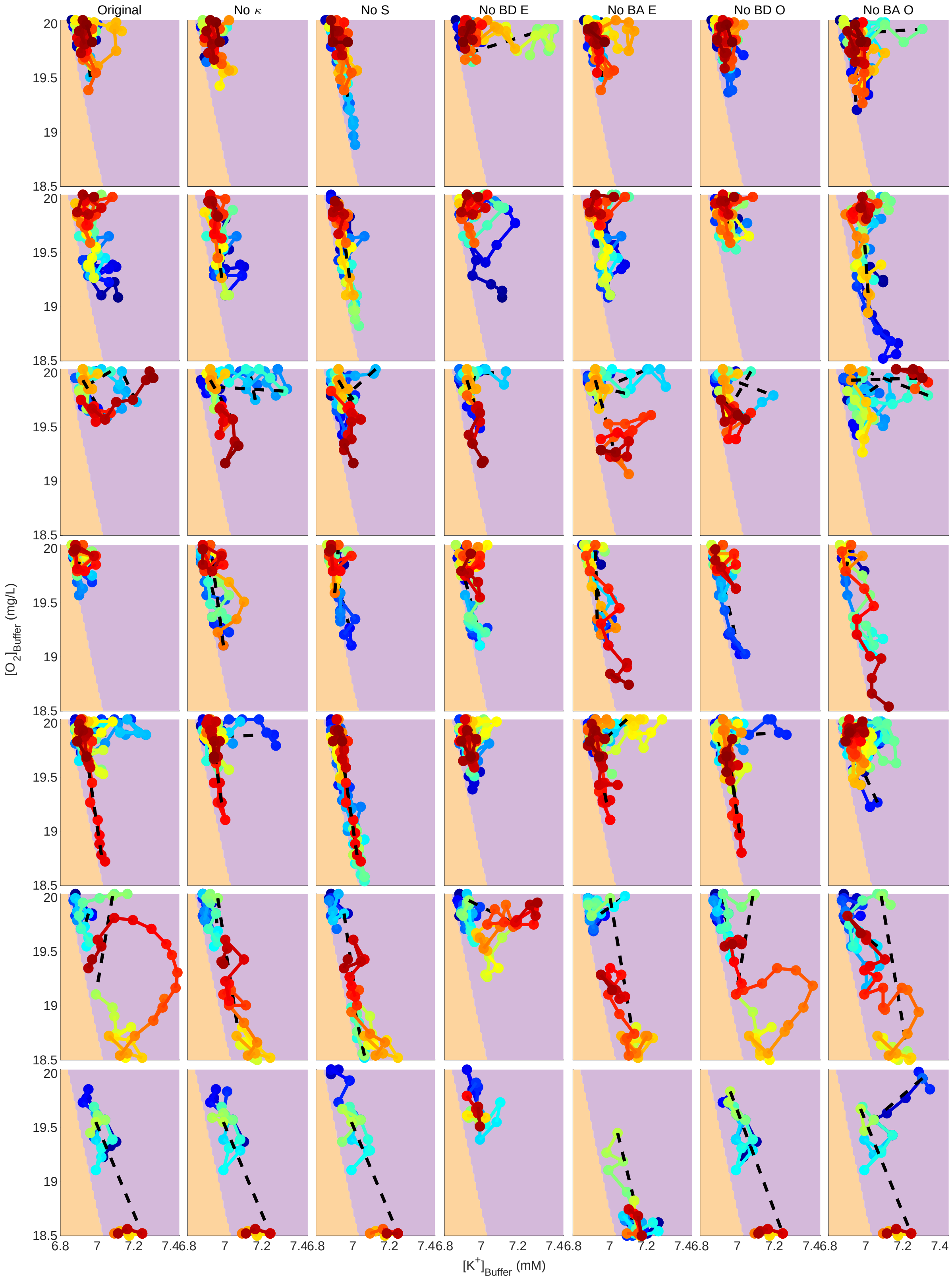

Supplementary Figure S10: Comparing the inferred parameters using all six burst metrics (original) with scenarios where one metric is deleted while keeping the other five, for infants with poor recovery outcomes. Shading is as per Fig. 4 in the main text.

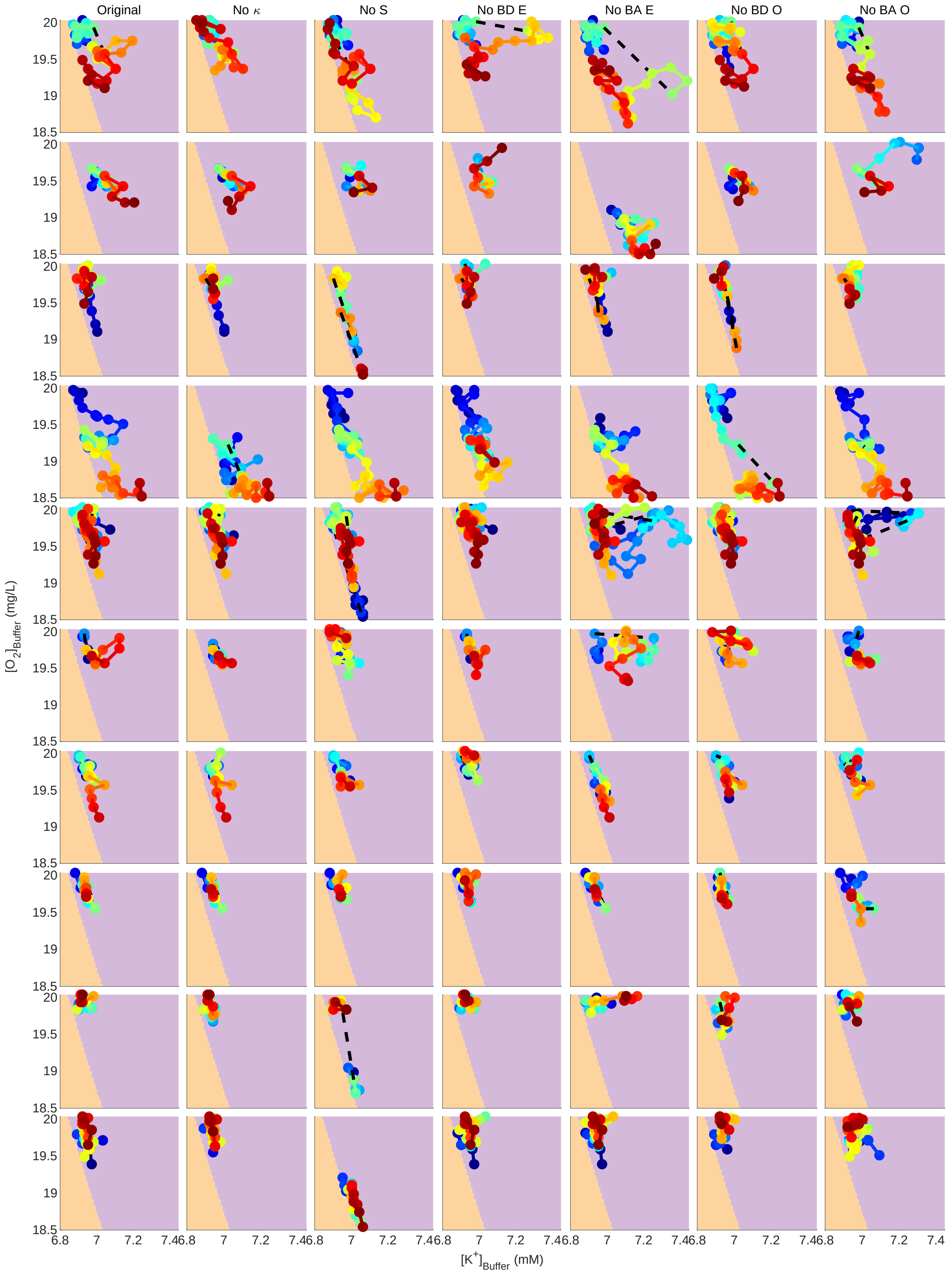

Supplementary Figure S11: Comparing the inferred parameters using all six burst metrics (original) with scenarios where one metric is deleted while keeping the other five, for infants with good recovery outcomes. Shading is as per Fig. 4 in the main text.

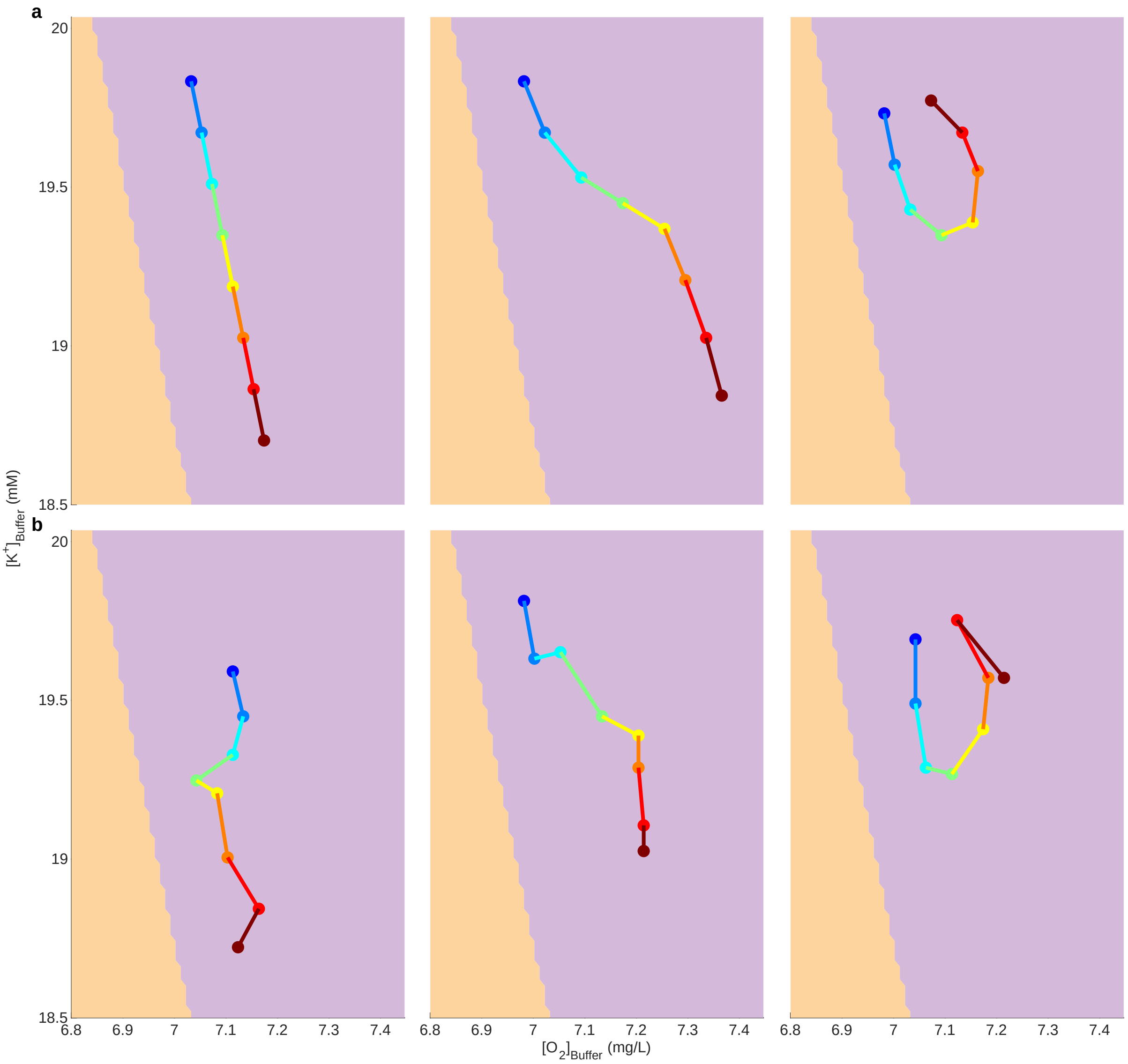

Supplementary Figure S12: Inferring back the parameters from simulated time-series. **a**, Three distinguishable synthetic trajectories. **b**, Inferred parameters from the simulated time series of the trajectory in panel a. Shading is as per Fig. 4 in the main text.

Supplementary movies 1 to 17 (attached)
