# Supplementary material for: Mechanisms underlying pathological cortical bursts during metabolic depletion
Source: Nat Commun. 2023 Aug 8;14:4792. doi: 10.1038/s41467-023-40437-0 (PMC10409751; doi:10.1038/s41467-023-40437-0)
Supplement: Supplementary file 3 — Description of Additional Supplementary Files [file 41467_2023_40437_MOESM3_ESM.pdf]

Description of Additional Supplementary Files

File Name: Supplementary Movie 1
Description: **Model generated trajectory, data, and burst statistics for infant 1 with poor outcome**. **a**, Inferred trajectories in the model parameter space. Colors denote time along the complete trajectories, from blue to red, with black dashed lines connecting epochs. Shading is as per Fig. 4 in the main text. **b**, Left panel shows the infant's time series for each epoch, arranged vertically. Right panel shows the model simulated time series using the inferred parameters in **a**. Green and pink boxes in the left and right panels, respectively, indicate 5 min windows analyzed in **c**. Color in the time series becomes warmer with time as per the accompanying trajectory in **a**. Black color represents the windows where the burst metrics could not be computed. **c**, Burst metrics of the windows highlighted in **b**, expanded here in the left panels (data in green, model in pink). Right panels show probability density functions (PDFs) for burst areas (BA) and durations (BD), along with average burst shapes.

File Name: Supplementary Movie 2
Description: **Model generated trajectory, data, and burst statistics for infant 2 with poor outcome**. **a**, Inferred trajectories in the model parameter space. Colors denote time along the complete trajectories, from blue to red, with black dashed lines connecting epochs. Shading is as per Fig. 4 in the main text. **b**, Left panel shows the infant's time series for each epoch, arranged vertically. Right panel shows the model simulated time series using the inferred parameters in **a**. Green and pink boxes in the left and right panels, respectively, indicate 5 min windows analyzed in **c**. Color in the time series becomes warmer with time as per the accompanying trajectory in **a**. Black color represents the windows where the burst metrics could not be computed. **c**, Burst metrics of the windows highlighted in **b**, expanded here in the left panels (data in green, model in pink). Right panels show probability density functions (PDFs) for burst areas (BA) and durations (BD), along with average burst shapes.

File Name: Supplementary Movie 3
Description: **Model generated trajectory, data, and burst statistics for infant 3 with poor outcome**. **a**, Inferred trajectories in the model parameter space. Colors denote time along the complete trajectories, from blue to red, with black dashed lines connecting epochs. Shading is as per Fig. 4 in the main text. **b**, Left panel shows the infant's time series for each epoch, arranged vertically. Right panel shows the model simulated time series using the inferred parameters in **a**. Green and pink boxes in the left and right panels, respectively, indicate 5 min windows analyzed in **c**. Color in the time series becomes warmer with time as per the accompanying trajectory in **a**. Black color represents the windows where the burst metrics could not be computed. **c**, Burst metrics of the windows highlighted in **b**, expanded here in the left panels (data in green, model in pink). Right panels show probability density functions (PDFs) for burst areas (BA) and durations (BD), along with average burst shapes.

File Name: Supplementary Movie 4
Description: **Model generated trajectory, data, and burst statistics for infant 4 with poor outcome**. **a**, Inferred trajectories in the model parameter space. Colors denote time along the complete trajectories, from blue to red, with black dashed lines connecting epochs. Shading is as per Fig. 4 in the main text. **b**, Left panel shows the infant's time series for each epoch, arranged

vertically. Right panel shows the model simulated time series using the inferred parameters in **a**. Green and pink boxes in the left and right panels, respectively, indicate 5 min windows analyzed in **c**. Color in the time series becomes warmer with time as per the accompanying trajectory in **a**. Black color represents the windows where the burst metrics could not be computed. **c**, Burst metrics of the windows highlighted in **b**, expanded here in the left panels (data in green, model in pink). Right panels show probability density functions (PDFs) for burst areas (BA) and durations (BD), along with average burst shapes.

File Name: Supplementary Movie 5
Description: **Model generated trajectory, data, and burst statistics for infant 5 with poor outcome**. **a**, Inferred trajectories in the model parameter space. Colors denote time along the complete trajectories, from blue to red, with black dashed lines connecting epochs. Shading is as per Fig. 4 in the main text. **b**, Left panel shows the infant's time series for each epoch, arranged vertically. Right panel shows the model simulated time series using the inferred parameters in **a**. Green and pink boxes in the left and right panels, respectively, indicate 5 min windows analyzed in **c**. Color in the time series becomes warmer with time as per the accompanying trajectory in **a**. Black color represents the windows where the burst metrics could not be computed. **c**, Burst metrics of the windows highlighted in **b**, expanded here in the left panels (data in green, model in pink). Right panels show probability density functions (PDFs) for burst areas (BA) and durations (BD), along with average burst shapes.

File Name: Supplementary Movie 6
Description: **Model generated trajectory, data, and burst statistics for infant 6 with poor outcome**. **a**, Inferred trajectories in the model parameter space. Colors denote time along the complete trajectories, from blue to red, with black dashed lines connecting epochs. Shading is as per Fig. 4 in the main text. **b**, Left panel shows the infant's time series for each epoch, arranged vertically. Right panel shows the model simulated time series using the inferred parameters in **a**. Green and pink boxes in the left and right panels, respectively, indicate 5 min windows analyzed in **c**. Color in the time series becomes warmer with time as per the accompanying trajectory in **a**. Black color represents the windows where the burst metrics could not be computed. **c**, Burst metrics of the windows highlighted in **b**, expanded here in the left panels (data in green, model in pink). Right panels show probability density functions (PDFs) for burst areas (BA) and durations (BD), along with average burst shapes.

File Name: Supplementary Movie 7
Description: **Model generated trajectory, data, and burst statistics for infant 7 with poor outcome**. **a**, Inferred trajectories in the model parameter space. Colors denote time along the complete trajectories, from blue to red, with black dashed lines connecting epochs. Shading is as per Fig. 4 in the main text. **b**, Left panel shows the infant's time series for each epoch, arranged vertically. Right panel shows the model simulated time series using the inferred parameters in **a**. Green and pink boxes in the left and right panels, respectively, indicate 5 min windows analyzed in **c**. Color in the time series becomes warmer with time as per the accompanying trajectory in **a**. Black color represents the windows where the burst metrics could not be computed. **c**, Burst metrics of the windows highlighted in **b**, expanded here in the left panels (data in green, model in pink). Right panels show probability density functions (PDFs) for burst areas (BA) and durations (BD), along with average burst shapes.

File Name: Supplementary Movie 8
Description: **Model generated trajectory, data, and burst statistics for infant 1 with good outcome**. **a**, Inferred trajectories in the model parameter space. Colors denote time along the complete trajectories, from blue to red, with black dashed lines connecting epochs. Shading is as per Fig. 4 in the main text. **b**, Left panel shows the infant's time series for each epoch, arranged vertically. Right panel shows the model simulated time series using the inferred parameters in **a**. Green and pink boxes in the left and right panels, respectively, indicate 5 min windows analyzed in **c**. Color in the time series becomes warmer with time as per the accompanying trajectory in **a**. Black color represents the windows where the burst metrics could not be computed. **c**, Burst metrics of the windows highlighted in **b**, expanded here in the left panels (data in green, model in pink). Right panels show probability density functions (PDFs) for burst areas (BA) and durations (BD), along with average burst shapes.

File Name: Supplementary Movie 9
Description: **Model generated trajectory, data, and burst statistics for infant 2 with good outcome**. **a**, Inferred trajectories in the model parameter space. Colors denote time along the complete trajectories, from blue to red, with black dashed lines connecting epochs. Shading is as per Fig. 4 in the main text. **b**, Left panel shows the infant's time series for each epoch, arranged vertically. Right panel shows the model simulated time series using the inferred parameters in **a**. Green and pink boxes in the left and right panels, respectively, indicate 5 min windows analyzed in **c**. Color in the time series becomes warmer with time as per the accompanying trajectory in **a**. Black color represents the windows where the burst metrics could not be computed. **c**, Burst metrics of the windows highlighted in **b**, expanded here in the left panels (data in green, model in pink). Right panels show probability density functions (PDFs) for burst areas (BA) and durations (BD), along with average burst shapes.

File Name: Supplementary Movie 10
Description: **Model generated trajectory, data, and burst statistics for infant 3 with good outcome**. **a**, Inferred trajectories in the model parameter space. Colors denote time along the complete trajectories, from blue to red, with black dashed lines connecting epochs. Shading is as per Fig. 4 in the main text. **b**, Left panel shows the infant's time series for each epoch, arranged vertically. Right panel shows the model simulated time series using the inferred parameters in **a**. Green and pink boxes in the left and right panels, respectively, indicate 5 min windows analyzed in **c**. Color in the time series becomes warmer with time as per the accompanying trajectory in **a**. Black color represents the windows where the burst metrics could not be computed. **c**, Burst metrics of the windows highlighted in **b**, expanded here in the left panels (data in green, model in pink). Right panels show probability density functions (PDFs) for burst areas (BA) and durations (BD), along with average burst shapes.

File Name: Supplementary Movie 11
Description: **Model generated trajectory, data, and burst statistics for infant 4 with good outcome**. **a**, Inferred trajectories in the model parameter space. Colors denote time along the complete trajectories, from blue to red, with black dashed lines connecting epochs. Shading is as per Fig. 4 in the main text. **b**, Left panel shows the infant's time series for each epoch, arranged vertically. Right panel shows the model simulated time series using the inferred parameters in **a**. Green and pink boxes in the left and right panels, respectively, indicate 5 min windows analyzed

in **c**. Color in the time series becomes warmer with time as per the accompanying trajectory in **a**. Black color represents the windows where the burst metrics could not be computed. **c**, Burst metrics of the windows highlighted in **b**, expanded here in the left panels (data in green, model in pink). Right panels show probability density functions (PDFs) for burst areas (BA) and durations (BD), along with average burst shapes.

File Name: Supplementary Movie 12
Description: **Model generated trajectory, data, and burst statistics for infant 5 with good outcome**. **a**, Inferred trajectories in the model parameter space. Colors denote time along the complete trajectories, from blue to red, with black dashed lines connecting epochs. Shading is as per Fig. 4 in the main text. **b**, Left panel shows the infant's time series for each epoch, arranged vertically. Right panel shows the model simulated time series using the inferred parameters in **a**. Green and pink boxes in the left and right panels, respectively, indicate 5 min windows analyzed in **c**. Color in the time series becomes warmer with time as per the accompanying trajectory in **a**. Black color represents the windows where the burst metrics could not be computed. **c**, Burst metrics of the windows highlighted in **b**, expanded here in the left panels (data in green, model in pink). Right panels show probability density functions (PDFs) for burst areas (BA) and durations (BD), along with average burst shapes.

File Name: Supplementary Movie 13
Description: **Model generated trajectory, data, and burst statistics for infant 6 with good outcome**. **a**, Inferred trajectories in the model parameter space. Colors denote time along the complete trajectories, from blue to red, with black dashed lines connecting epochs. Shading is as per Fig. 4 in the main text. **b**, Left panel shows the infant's time series for each epoch, arranged vertically. Right panel shows the model simulated time series using the inferred parameters in **a**. Green and pink boxes in the left and right panels, respectively, indicate 5 min windows analyzed in **c**. Color in the time series becomes warmer with time as per the accompanying trajectory in **a**. Black color represents the windows where the burst metrics could not be computed. **c**, Burst metrics of the windows highlighted in **b**, expanded here in the left panels (data in green, model in pink). Right panels show probability density functions (PDFs) for burst areas (BA) and durations (BD), along with average burst shapes.

File Name: Supplementary Movie 14
Description: **Model generated trajectory, data, and burst statistics for infant 7 with good outcome**. **a**, Inferred trajectories in the model parameter space. Colors denote time along the complete trajectories, from blue to red, with black dashed lines connecting epochs. Shading is as per Fig. 4 in the main text. **b**, Left panel shows the infant's time series for each epoch, arranged vertically. Right panel shows the model simulated time series using the inferred parameters in **a**. Green and pink boxes in the left and right panels, respectively, indicate 5 min windows analyzed in **c**. Color in the time series becomes warmer with time as per the accompanying trajectory in **a**. Black color represents the windows where the burst metrics could not be computed. **c**, Burst metrics of the windows highlighted in **b**, expanded here in the left panels (data in green, model in pink). Right panels show probability density functions (PDFs) for burst areas (BA) and durations (BD), along with average burst shapes.

File Name: Supplementary Movie 15
Description: **Model generated trajectory, data, and burst statistics for infant 8 with good outcome**. **a**, Inferred trajectories in the model parameter space. Colors denote time along the complete trajectories, from blue to red, with black dashed lines connecting epochs. Shading is as per Fig. 4 in the main text. **b**, Left panel shows the infant's time series for each epoch, arranged vertically. Right panel shows the model simulated time series using the inferred parameters in **a**. Green and pink boxes in the left and right panels, respectively, indicate 5 min windows analyzed in **c**. Color in the time series becomes warmer with time as per the accompanying trajectory in **a**. Black color represents the windows where the burst metrics could not be computed. **c**, Burst metrics of the windows highlighted in **b**, expanded here in the left panels (data in green, model in pink). Right panels show probability density functions (PDFs) for burst areas (BA) and durations (BD), along with average burst shapes.

File Name: Supplementary Movie 16
Description: **Model generated trajectory, data, and burst statistics for infant 9 with good outcome**. **a**, Inferred trajectories in the model parameter space. Colors denote time along the complete trajectories, from blue to red, with black dashed lines connecting epochs. Shading is as per Fig. 4 in the main text. **b**, Left panel shows the infant's time series for each epoch, arranged vertically. Right panel shows the model simulated time series using the inferred parameters in **a**. Green and pink boxes in the left and right panels, respectively, indicate 5 min windows analyzed in **c**. Color in the time series becomes warmer with time as per the accompanying trajectory in **a**. Black color represents the windows where the burst metrics could not be computed. **c**, Burst metrics of the windows highlighted in **b**, expanded here in the left panels (data in green, model in pink). Right panels show probability density functions (PDFs) for burst areas (BA) and durations (BD), along with average burst shapes.

File Name: Supplementary Movie 17
Description: **Model generated trajectory, data, and burst statistics for infant 10 with good outcome**. **a**, Inferred trajectories in the model parameter space. Colors denote time along the complete trajectories, from blue to red, with black dashed lines connecting epochs. Shading is as per Fig. 4 in the main text. **b**, Left panel shows the infant's time series for each epoch, arranged vertically. Right panel shows the model simulated time series using the inferred parameters in **a**. Green and pink boxes in the left and right panels, respectively, indicate 5 min windows analyzed in **c**. Color in the time series becomes warmer with time as per the accompanying trajectory in **a**. Black color represents the windows where the burst metrics could not be computed. **c**, Burst metrics of the windows highlighted in **b**, expanded here in the left panels (data in green, model in pink). Right panels show probability density functions (PDFs) for burst areas (BA) and durations (BD), along with average burst shapes.
