# Supplementary material for: Mechanisms underlying pathological cortical bursts during metabolic depletion
Source: Nat Commun. 2023 Aug 8;14:4792. doi: 10.1038/s41467-023-40437-0 (PMC10409751; doi:10.1038/s41467-023-40437-0)
Supplement: Supplementary file 21 — Reporting Summary [file 41467_2023_40437_MOESM21_ESM.pdf]

nature portfolio

Corresponding author(s): SD

Last updated by author(s): Jun 18, 2023

Reporting Summary

Nature Portfolio wishes to improve the reproducibility of the work that we publish. This form provides structure for consistency and transparency in reporting. For further information on Nature Portfolio policies, see our Editorial Policies and the Editorial Policy Checklist.

Statistics

For all statistical analyses, confirm that the following items are present in the figure legend, table legend, main text, or Methods section.

n/a	Confirmed	
☐	☒	The exact sample size (*n*) for each experimental group/condition, given as a discrete number and unit of measurement
☐	☒	A statement on whether measurements were taken from distinct samples or whether the same sample was measured repeatedly
☐	☒	The statistical test(s) used AND whether they are one- or two-sided<br>*Only common tests should be described solely by name; describe more complex techniques in the Methods section.*
☒	☐	A description of all covariates tested
☒	☐	A description of any assumptions or corrections, such as tests of normality and adjustment for multiple comparisons
☐	☒	A full description of the statistical parameters including central tendency (e.g. means) or other basic estimates (e.g. regression coefficient) AND variation (e.g. standard deviation) or associated estimates of uncertainty (e.g. confidence intervals)
☐	☒	For null hypothesis testing, the test statistic (e.g. *F*, *t*, *r*) with confidence intervals, effect sizes, degrees of freedom and *P* value noted<br>*Give P values as exact values whenever suitable.*
☒	☐	For Bayesian analysis, information on the choice of priors and Markov chain Monte Carlo settings
☒	☐	For hierarchical and complex designs, identification of the appropriate level for tests and full reporting of outcomes
☒	☐	Estimates of effect sizes (e.g. Cohen's *d*, Pearson's *r*), indicating how they were calculated

Our web collection on statistics for biologists contains articles on many of the points above.

Software and code

Policy information about availability of computer code

Data collection	No data was collected in this study.
Data analysis	Custom-written computer codes (MATLAB 2021b) for generating raw and processed simulation data are accessible via DOI https://doi.org/10.5281/zenodo.8013692. DP algorithm for estimating trajectories was developed in this study, and its code is accessible via DOI https://doi.org/10.5281/zenodo.8013692. For burst extraction we used the method developed in Roberts, J. A., Iyer, K. K., Finnigan, S., Vanhatalo, S. & Breakspear, M. Scale-free bursting in human cortex following hypoxia at birth. Journal of neuroscience 34, 6557–6572 (2014). To estimate the power-law fits, we used the method developed in Deluca, A., Corral, Á. Fitting and goodness-of-fit test of non-truncated and truncated power-law distributions. Acta Geophys. 61, 1351–1394 (2013).

For manuscripts utilizing custom algorithms or software that are central to the research but not yet described in published literature, software must be made available to editors and reviewers. We strongly encourage code deposition in a community repository (e.g. GitHub). See the Nature Portfolio guidelines for submitting code & software for further information.

Data

Policy information about availability of data

All manuscripts must include a data availability statement. This statement should provide the following information, where applicable:

- Accession codes, unique identifiers, or web links for publicly available datasets
- A description of any restrictions on data availability
- For clinical datasets or third party data, please ensure that the statement adheres to our policy

The raw and processed simulation data generated in this study, which are plotted in the figures, have been deposited in the Figshare database, accessible via DOI https://doi.org/10.6084/m9.figshare.23514531.v1. The EEG data from human infants are sensitive data that cannot be distributed without pertinent preprocessing to ensure anonymity as well as relevant data sharing agreements with Helsinki University Hospital (via author S.V.). However, the anonymized analytic derivative of this EEG data (EEG power, such as in Fig. 2a-d) has been deposited in the Figshare database, accessible via the same DOI https://doi.org/10.6084/m9.figshare.23514531.v1.

Human research participants

Policy information about studies involving human research participants and Sex and Gender in Research.

Reporting on sex and gender	We have been careful to avoid confusing sex and gender; for newborn infants, data on gender is not available [or even gender is ill-defined at that age]. Sex was determined from the clinical records, where it was assigned by the treating clinicians at birth. We do not have consent to share individual-level data and so do not provide disaggregated sex data. No sex- and gender-based analyses have been performed. Our study is primarily a modelling study seeking to describe general features of human neurophysiology; a proof-of-principle application of the model to human data uses a convenience sample of newborn infants that is not adequately powered to perform sex- or gender-based analysis.
Population characteristics	We used existing data from 17 infants recovering from birth asphyxia to infer the generative model's parameters. This was an existing archived dataset.
Recruitment	Participants were infants, and were admitted to neonatal intensive care unit based on whether they suffered from birth asphyxia.
Ethics oversight	The use of the retrospectively collected, archived patient data was approved by the Ethics Committee of the Hospital for Children and Adolescents, Helsinki University Central Hospital

Note that full information on the approval of the study protocol must also be provided in the manuscript.

Field-specific reporting

Please select the one below that is the best fit for your research. If you are not sure, read the appropriate sections before making your selection.

☒ Life sciences ☐ Behavioural & social sciences ☐ Ecological, evolutionary & environmental sciences

For a reference copy of the document with all sections, see nature.com/documents/nr-reporting-summary-flat.pdf

Life sciences study design

All studies must disclose on these points even when the disclosure is negative.

Sample size	This is predominantly a computational modeling study that used retrospective datasets with no prior determination of sample size. The datasets included scalp-EEG from a cohort of 17 infants recovering form birth asphyxia with 2 year outcomes grouped as good outcome and poor outcome. Some data within these cohorts have been previously published (Roberts et al. 2014a; Iyer et al. 2014). The scope of these previous works was independent of the current study.
Data exclusions	No data exclusions
Replication	To ensure that our findings can be reliably reproduced, our codes and data will be available upon request.
Randomization	Network connectivity between neurons was randomized; there was no other randomization in this study.
Blinding	Investigators were not blinded to any aspect of this study.

Reporting for specific materials, systems and methods

We require information from authors about some types of materials, experimental systems and methods used in many studies. Here, indicate whether each material, system or method listed is relevant to your study. If you are not sure if a list item applies to your research, read the appropriate section before selecting a response.

Materials & experimental systems

n/a	Involved in the study
☒	☐ Antibodies
☒	☐ Eukaryotic cell lines
☒	☐ Palaeontology and archaeology
☒	☐ Animals and other organisms
☒	☐ Clinical data
☒	☐ Dual use research of concern

Methods

n/a	Involved in the study
☒	☐ ChIP-seq
☒	☐ Flow cytometry
☒	☐ MRI-based neuroimaging
